# LI-AGCN: A Lightweight Initialization-Enhanced Adaptive Graph Convolutional Network for Effective Skeleton-Based Action Recognition

**DOI:** 10.3390/s25237282

**Published:** 2025-11-29

**Authors:** Qingsheng Xie, Hongmin Deng

**Affiliations:** College of Electronics and Information Engineering, Sichuan University, No. 24, Section 1, Yihuan Road, Wuhou District, Chengdu 610065, China; 2023222050063@stu.scu.edu.cn

**Keywords:** skeleton-based action recognition, graph convolutional network, lightweight, initialization-enhanced model, coordinate-based branch

## Abstract

The graph convolutional network (GCN) has become a mainstream technology in skeleton-based action recognition since it was first applied to this field. However, previous studies often overlooked the pivotal role of heuristic model initialization in the extraction of spatial features, impeding the model from achieving its optimal performance. To address this issue, a lightweight initialization-enhanced adaptive graph convolutional network (LI-AGCN) is proposed, which effectively captures spatiotemporal features while maintaining low computational complexity. LI-AGCN employs three coordinate-based input branches (CIB) to dynamically adjust graph structures, which facilitates the extraction of informative spatial features. In addition, the model incorporates a lightweight and multi-scale temporal module to extract temporal feature, and employs an attention module that considers the temporal, spatial, and channel dimensions simultaneously to enhance key features. Finally, the performance of our proposed model is evaluated on three large-scale public datasets: NTU RGB+D, NTU RGB+D 120, and UAV-Human. The experimental results demonstrate that the LI-AGCN achieves excellent comprehensive performances on these datasets, especially obtaining 90.03% accuracy on the cross-subject benchmark of the NTU RGB+D dataset with only 0.18 million parameters, showcasing the effectiveness of the model.

## 1. Introduction

Human action recognition (HAR) is a crucial task in the field of computer vision and has garnered significant attention in recent years. The primary goal of HAR is to automatically recognize the behavior categories of individuals or groups by analyzing video or sensor data. This research has vital applications in several areas such as video surveillance [1], human–computer interaction [2], and virtual reality [3].

Traditional methods for action recognition primarily relied on RGB videos or depth sequences. However, RGB images were highly sensitive to environmental factors such as lighting and background variations, while depth images performed poorly under long-range or occluded conditions. In contrast, skeleton data captured the coordinates of key human joints, providing a more robust representation of the human body’s structure and dynamic features. As a result, skeleton data has become an essential data source for action recognition research in recent years.

Skeleton-based action recognition methods have evolved through two primary phases. Early works predominantly utilized convolutional neural networks (CNNs) [4,5,6,7] and recurrent neural networks (RNNs) [8,9,10] to extract spatial and temporal features from skeleton sequences. While CNNs exhibited limitations in handling temporal data, RNNs faced challenges in capturing global dependencies over long sequences due to high computational complexity. In recent years, graph convolutional networks (GCNs) have emerged as an effective tool for processing non-Euclidean data, being especially popular in skeleton-based action recognition. GCNs can naturally describe the topological structure of human joints and effectively capture dependencies between joints, as a crucial model for action recognition tasks. Yan et al. [11] first proposed ST-GCN for action recognition; since then, GCN-based methods have become the mainstream approach for constructing action recognition models.

**Figure 1:**
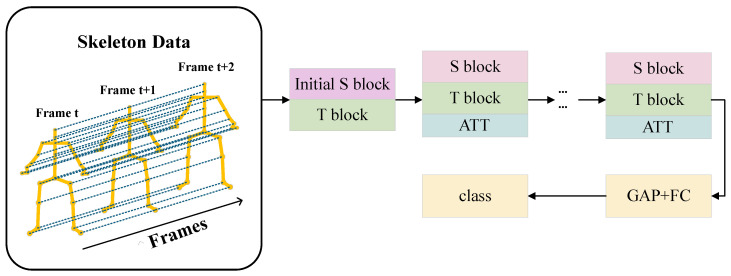
The simple structure diagram of LI-AGCN, where GAP and FC represent the global average pooling operation and fully connected layer, respectively, and S block and T block represent the spatial block and temporal block, respectively.

Current action recognition models often need high parameter count and floating-point operations (FLOPs), resulting in increased resource usage and slower inference speeds, posing challenges for real-world deployment. To tackle this challenge, we propose a novel model named lightweight initialization-enhanced adaptive graph convolution network (LI-AGCN), which incorporates the coordinate-based input branches (CIB) architecture. Different from the multi-stream strategy in many previous works [12,13,14,15,16,17], the CIB architecture uniquely divides all raw skeleton sequence information into independent input streams in three coordinate axes, rather than in different features or modalities. This design significantly reduces model parameter count and computational complexity while maintaining competitive performance. The simplified structure diagram of the LI-AGCN is shown in Figure 1.

Most existing GCN-based action recognition methods underemphasized the first spatial feature extraction from initial skeleton data. Typically, GCNs directly extracted spatial features from raw skeleton data, followed by CNNs or RNNs to capture temporal information. However, the lack of targeted optimization during the spatial feature extraction in the model’s initialization phase often limits the feature expression capabilities in subsequent layers. Current spatial initialization modules tended to be simplistic, making the effectiveness of spatial feature extraction a key determinant of overall network performance. Additionally, traditional spatial feature extraction methods often largely relied on predefined graph structures, aggregating and updating joints based on the skeleton’s adjacency matrix. These approaches undervalued potential dynamic relationships between nodes, limiting their ability to capture flexible interactions in action sequences. In this paper, we design the action-driven spatial relation adaptive graph convolutional (ASR-AGC) module, which can extract richer spatial information from raw skeleton sequences.

Regarding temporal feature extraction, prior research explored optimization from both lightweight and multi-scale perspectives. For instance, ResGCN [12] reduced computational complexity by employing bottleneck structures [18]. Building upon this, EfficientGCN [13] further enhanced the design through a compound expansion strategy, replacing bottleneck structures with modified versions of SepLayer, EpSepLayer, and SGLayer. These layers were inspired by three distinct implementations of separable convolutions [19,20,21]. From a multi-scale perspective, MS-G3D [22] employed multi-scale strategies to capture both short-term and long-term temporal dependencies through various temporal windows, thereby improving the representation of complex temporal relationships. To leverage the advantages of both lightweight and multi-scale approaches, this study designs the multi-scale temporal feature fusion (MSTFF) module. The MSTFF module integrates lightweight design principles and multi-scale modeling techniques to effectively capture short-term dynamics and long-term dependencies in temporal features.

In skeleton-based action recognition, attention modules played a vital role in enhancing the representation of key features. Existing works, such as ST-JointAtt [13] and SE-Net [23], explored attention mechanisms to focus on important dimensions. ST-JointAtt integrated temporal and spatial attention to improve feature modeling, while SE-Net enhanced channel-wise information. This paper designs a unified attention module named channel-enhanced spatial temporal joint attention (CEST-JAtt), which integrates temporal, spatial, and channel dimensions into a cohesive framework, enabling a more comprehensive representation of multi-dimensional features.

The main contributions of this paper are summarized as follows:

- The novel LI-AGCN model with CIB architecture is proposed, which includes three key modules: ASR-AGC, MSTFF, and CEST-JAtt modules. The LI-AGCN model significantly reduces model parameter count and computational complexity.
- The ASR-AGC module is designed to capture dynamic relationships between joints more effectively for better initial spatial feature extraction; the MSTFF module is designed to leverage lightweight and multi-scale strategies through the use of dilated convolutions and group convolutions; the CEST-JAtt module is designed to unify attention mechanisms across temporal, spatial, and channel dimensions, enabling comprehensive feature interrelationship modeling and enhancing overall network representation capabilities.
- Extensive experiments on LI-AGCN model are conducted, demonstrating superior performance and efficiency on three large-scale action recognition datasets (NTU RGB+D, NTU RGB+D 120, UAV-Human).

## 2. Related Work

### 2.1. GCNs for Skeleton-Based Action Recognition

The application of GCNs to skeleton-based action recognition was pioneered by Yan et al. [11] through the introduction of ST-GCN. This approach captured spatial and temporal relationships among skeletal joints, significantly improving recognition accuracy. The original ST-GCN introduced three adjacency partitioning strategies for joint nodes, which were followed by numerous innovative partitioning approaches [24,25]. Subsequently, Shi et al. [26] proposed 2s-AGCN, which utilized a dual-stream structure to separately process spatial and temporal information in skeleton data, enhancing the model’s ability to recognize complex actions.

Chen et al. [27] further advanced this field by proposing CTR-GCN to learn diverse topological structures and effectively aggregate joint features. Liu et al. [22] introduced MS-G3D, which extended the domain of action recognition by integrating 3D graph convolutions to achieve unified spatiotemporal modeling, thereby increasing feature diversity and significantly improving recognition accuracy. This approach inspired further research on spatiotemporal fusion [24,28].

Additionally, some works explored multi-modal data fusion. For instance, Song et al. [29] combined skeleton data with RGB images, fusing the extracted features from both types of data to enhance recognition performance. Although these methods achieved significant improvements in recognition accuracy, they often increased computational complexity, posing challenges for practical deployment.

### 2.2. Lightweight Models

The parameter count and computational complexity are critical performance indicators for action recognition models, directly influencing their suitability for deployment on edge computing devices. In recent years, many efforts have been made to optimize these metrics. For instance, EfficientGCN [13] proposed multiple lightweight structures and applied a compound scaling strategy to efficiently develop GCN baselines with low parameter count and high accuracy. In addition, a lightweight deep model based on knowledge distillation [30] was proposed to address power consumption and computational constraints on edge devices.

These advancements in lightweight design improved the adaptability of action recognition models to resource-constrained environments, such as Internet of Things (IoT) applications. However, lightweight models often face trade-offs between reducing complexity and maintaining performance, necessitating a careful balance to ensure efficient inference without compromising recognition accuracy.

### 2.3. Attention Mechanisms

Attention mechanisms were widely applied in computer vision tasks to enhance the focus on important features, thereby improving recognition accuracy. Classic attention mechanisms, such as SENet [23] and CBAM [31], enhanced critical features by weighting across different channels. Inspired by the Transformer [32] from the natural language processing (NLP) domain, which excelled in global feature modeling, many recent works have integrated self-attention mechanisms into computer vision models [28,33,34,35]. MS-AAGCN [36] leveraged attention mechanisms across temporal, joint, and channel dimensions to independently enhance multi-dimensional features, thereby improving the model’s feature representation capabilities. Another notable approach was the Part-Att attention mechanism introduced by ResGCN [12], which divided human joints into several parts and applied weighted attention to enhance local feature extraction. Additionally, Li et al. [37] and Si et al. [38] utilized LSTM networks to extract temporal information, while EfficientGCN [13] introduced the ST-JointAtt to apply synchronous weighting across temporal and spatial dimensions, improving the recognition of complex actions. Xia et al. [39] further introduced two innovative self-constructed attention modules that focus on spatiotemporal features and channel dimension, respectively. Chen et al. [40] proposed a novel global attention mechanism that incorporates both spatial and temporal attention.

With the widespread application of attention mechanisms in action recognition, fine-grained modeling of multi-dimensional features has gradually become a key direction for improving recognition performance. A promising future direction involves combining channel dimension weighting with synchronized spatiotemporal feature processing, potentially leading to breakthroughs in optimizing attention mechanisms.

## 3. Methodology

In this section, the design and implementation of the proposed LI-AGCN for skeleton-based action recognition are systematically presented, which includes the CIB architecture and three key modules: ASR-AGC, MSTFF, and CEST-JAtt. Firstly, the principle of the GCN is reviewed. Secondly, the CIB structure is introduced in detail, which leverages an innovative multi-stream decomposition design, laying a solid foundation for feature extraction in the initial stage. Thirdly, the design principle and procedure of the ASR-AGC module are elaborated, which can enhance the extraction of spatial features during the initial stage and capture dynamic dependencies. Fourthly, the MSTFF module is discussed, which combines lightweight and multi-scale modeling strategies to optimize the capturing and fusion of temporal features. Finally, the designed CEST-JAtt module simultaneously assigns different weights to the features in the temporal, spatial, and channel dimensions, achieving comprehensive multi-dimensional feature fusion and efficient representation. This significantly enhances the network’s feature learning capability and robustness.

**Figure 2:**
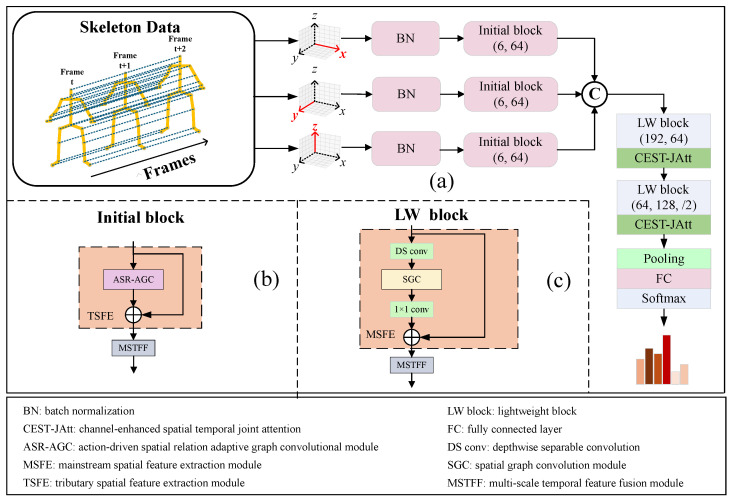
(**a**) The detailed architecture of LI-AGCN, where ⓒ denotes concatenation operation along the channel dimension. The numbers inside the Initial block and LW block indicate the number of input and output channels, respectively, while /2 signifies a temporal stride of 2. (**b**) The detailed structure of Initial block. (**c**) The detailed structure of LW block, where ⊕ in (**b**,**c**) represents element-wise addition operation.

The multi-stream input, processing pipeline, and the feature extraction mechanism after fusion in the complete LI-AGCN are illustrated in Figure 2a. LI-AGCN processes three skeleton sequences of dimensions *C* × *T* × *V*, corresponding to the number of channels, temporal frames, and joints, respectively. The data first passes through a batch normalization layer, followed by an initial block that expands the channel dimension to 64 (detailed in Figure 2b). The data from the three streams are then concatenated along the channel dimension. A series of LW blocks further refine the features (detailed in Figure 2c), followed by global pooling. The aggregated features are finally fed into a fully-connected layer and a Softmax classifier for action prediction.

### 3.1. The Principle Review of GCN

The GCN approach represents the human skeleton as a graph structure, with joints as nodes and the natural bone connections forming the edges. This graph-based representation is particularly effective for processing skeletal data by preserving its inherent connectivity. Typically, an input skeleton sequence is structured as a third-order tensor of dimensions *C* × *T* × *V*, corresponding to the number of channels, temporal frames, and joints, respectively. Building upon the ST-GCN [11] framework, the spatial graph convolution at a given time step is mathematically defined as follows:

(1) $$f_{\mathrm{out}}=\sum_{k=0}^{K_{S}}W_{k}\left(f_{\mathrm{in}}A_{k}\right)⊙M_{k}$$

where $f_{\mathrm{in}}$ and $f_{\mathrm{out}}$ represent the input and output features maps; $K_{s}$ is a predefined maximum graph distance; $A_{k}=\Lambda_{k}^{-\frac{1}{2}}\bar{A_{k}}\Lambda_{k}^{-\frac{1}{2}}$, where ${\bar{A}}_{k}$ represents the *k*-th order adjacency matrix that marks the pairs of joints with a graph distance *k*; $\Lambda_{k}$ is used to normalize $\bar{A_{k}}$; $W_{k}\left(\cdot \right)$ is a learnable weight function; $M_{k}$ is an N×N attention map that indicates the importance of each vertex; ⊙ denotes the Hadamard product.

To capture dynamic motion patterns, temporal dependencies are modeled along the time axis. This is typically achieved by applying a standard 1D convolution with a kernel size of $K_{T}\times 1$ to the features of each joint across consecutive frames. The combination of spatial graph convolutions and temporal convolutions forms a powerful spatiotemporal architecture for skeleton-based action recognition.

### 3.2. CIB

#### 3.2.1. Model Architecture

As illustrated in Figure 2, the CIB architecture intuitively demonstrates the design of multi-stream coordinate axis inputs. The parts highlighted in blush pink in Figure 2b,c correspond to the tributary spatial feature extraction (TSFE) module and the mainstream spatial feature extraction (MSFE) module, respectively. Each input stream corresponds to a coordinate axis dimension, which integrates features such as position, velocity, and bone geometry, laying a solid foundation for feature representation. To achieve a lightweight model design, attention modules are not introduced during the multi-stream processing stage. Instead, attention mechanisms are incorporated only after the multi-stream fusion, employing an early fusion strategy. This approach enhances feature representation while maintaining model efficiency. Additionally, in the MSFE module, depthwise separable convolutions are employed for dimensionality reduction prior to feature extraction. This significantly reduces the model’s parameter count and computational complexity. This architecture not only preserves the abundance of input features but also drastically reduces model complexity, facilitating a smoother convergence during the training process.

#### 3.2.2. Data Preprocessing

The data preprocessing in this paper involves transforming the raw skeleton sequence into three distinct features: position, velocity, and bone geometry. These features are then restructured into three streams, corresponding to the x, y, and z axes. Each stream undergoes spatial and temporal feature extraction independently, followed by an early fusion process to combine the extracted features.

In a three-dimensional coordinate space, we assume that the skeleton sequence is represented as $x\in R^{C\times T\times V}$, where C=3 indicates the number of coordinate dimensions, *T* is the number of frames, and *V* is the number of joints.

**Joint Position:** The joint positions include absolute and relative position. The absolute positions *X* correspond to the original skeleton sequence *x*, i.e., X=x.

The relative positions of each joint are defined with respect to the coordinate of the central spine joint. The set of relative positions can be expressed as $P=\{p_{i}|i=1,2,\cdots ,V\}$, where $p_{i}$ is calculated using Equation (2):

(2) $$p_{i}=x\left[:,:,i\right]-x\left[:,:,s\right]$$

where *s* denotes the index of the central spine joint.

**Joint Velocity:** We define the set of velocities as $S=\{s_{t}|t=1,2,\cdots ,T\}$ for slow velocities and $F=\{f_{t}|t=1,2,\cdots ,T\}$ for fast velocities. The calculations for $s_{t}$ and $f_{t}$ are given by Equations (3) and (4):

(3) $$s_{t}=x\left[:,t+1,:\right]-x\left[:,t,:\right]$$

(4) $$f_{t}=x\left[:,t+2,:\right]-x\left[:,t,:\right]$$

**Bone Geometric Features:** The bone features include bone lengths and angles, denoted by $L=\{l_{i}|i=1,2,\cdots ,V\}$ and $A=\{a_{i}|i=1,2,\cdots ,V\}$, respectively. The bone length $l_{i}$ and bone angle $a_{i}$ are computed using Equations (5) and (6):

(5) $$l_{i}=x\left[:,:,i\right]-x\left[:,:,i_{adj}\right]$$

(6) $$a_{i}\left[j,:,:\right]=\arccos\left(\frac{l_{i}\left[j,:,:\right]}{\sqrt{l_{i}^{2}\left[1,:,:\right]+l_{i}^{2}\left[2,:,:\right]+l_{i}^{2}\left[3,:,:\right]}}\right)$$

where $i_{adj}$ represents the adjacent joint of the joint *i*, and j∈{1,2,3} refers to the three-dimensional coordinate system.

**Coordinate Stream Fusion of Features:** The features *X*, *P*, *S*, *F*, *L*, and *A* are concatenated along the coordinate dimensions *x*, *y*, and *z* to form three input streams, processed as follows in Equations (7)–(9):

(7) $$\begin{array}{cc} C_{x} & =X[1,:,:]||P[1,:,:]||S[1,:,:]\\ \; & \;||F[1,:,:]||L[1,:,:]||A[1,:,:] \end{array}$$

(8) $$\begin{array}{cc} C_{y} & =X[2,:,:]||P[2,:,:]||S[2,:,:]\\ \; & \;||F[2,:,:]||L[2,:,:]||A[2,:,:] \end{array}$$

(9) $$\begin{array}{cc} C_{z} & =X[3,:,:]||P[3,:,:]||S[3,:,:]\\ \; & \;||F[3,:,:]||L[3,:,:]||A[3,:,:] \end{array}$$

where || denotes the concatenation operation along the channel dimension, and $C_{x}\in R^{6\times T\times V}$, $C_{y}\in R^{6\times T\times V}$, $C_{z}\in R^{6\times T\times V}$.

CIB is an innovative architecture first proposed in this work, mainly to provide a suitable backbone for the ASR-AGC module and an alternative design option for future research, rather than to replace existing architectures.

### 3.3. ASR-AGC

**Figure 3:**
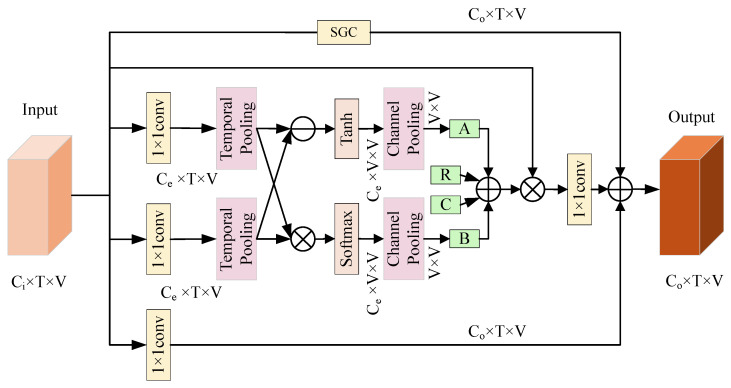
The structure of the ASR-AGC module, where ⊕ and ⊖ represent element-wise addition and element-wise subtraction, respectively; ⊗ represents matrix multiplication; and matrices *A*, *B*, *C*, and *R* refer to four weighted relational matrices that have the same shape as the adjacency matrix of the original skeleton graph.

As illustrated in Figure 3, the ASR-AGC module is designed to perform adaptive spatial graph convolution on skeleton data. Traditional spatial graph convolution relies on a predefined graph adjacency matrix, where the connections between any two nodes depend solely on physical connections. However, in certain actions, there may be strong correlations between nodes that are directly connected physically. Sole reliance on the original adjacency matrix can cause such potential relationships to be overlooked, thus limiting the network’s ability to learn spatial structures.

To address this issue, we design the ASR-AGC module, which utilizes adaptive graph convolution to dynamically make up the limitations of predefined graph convolutions. The ASR-AGC module enhances the modeling of relationships between arbitrary joint nodes through a composite adjacency matrix, which combines four matrices (*A*, *B*, *C*, and *R*) with corresponding weight coefficients. Matrices A and B, derived from input skeletal sequence through self-attention mechanisms, capture similarity information between arbitrary joint points. Matrix *C*, initialized as an identity matrix, reinforces self-node information, while matrix *R* encodes the relationship intensity between joint nodes by constructing a new relationship graph. The module is implemented as a residual branch to maintain model stability, with its computational process formulated in Equation (10):

(10) $$f_{out}=\left(f_{in}⊗\left(\alpha \cdot A+\beta \cdot B+B_{N}\left(C+R\right)\right)\right)⊙W+f_{SGC}$$

where $B_{N}$ denotes the normalization operation, *W* represents the weight function, and ⊗ and ⊙ represent matrix multiplication and element-wise multiplication, respectively. $f_{SGC}$ corresponds to $f_{out}$ in Equation (1). The specific definitions and calculations for *A*, *B*, *C*, and *R* are as follows. The shape of the input tensor is given by $x\in R^{3\times T\times V}$.

#### 3.3.1. Part 1 (*R*)

The value of *R* is a joint relationship metric that varies depending on the skeleton sequence. The calculation principle is as follows: first, the absolute value of the slow velocity obtained from Equation (3) is computed, and then summed along the temporal dimension. This reflects the temporal cumulative path length for each joint, which reflects the level of activity of the joint. A larger path length indicates a more active joint, while a smaller path length indicates the opposite.

Taking the *x* axis dimension as an example, the formula for calculating the path length of joints in the *x*-dimension is given by Equation (11):

(11) $$D_{x}=N_{1}\left(\sum_{t=1}^{T-1}\left|C_{x}\left[3,t,:\right]\right|\right)$$

where $C_{x}$ refers to the value obtained from Equation (7), $N_{1}$ denotes the min–max normalization operation, and |·| represents taking the absolute value, $D_{x}\in R^{V}$.

Subsequently, the difference in relative position vectors over time between each pair of joints is computed, then the magnitude of the difference vector is taken to sum along the temporal dimension. The resulting value reflects the inter-joint interaction intensity, which indicates the degree of closeness between two joints. A higher interaction intensity between any two joints implies a closer relationship, meaning that during graph convolution aggregation, nodes with higher interaction intensity will be assigned greater weights when aggregating features from other joints.

**Figure 4:**
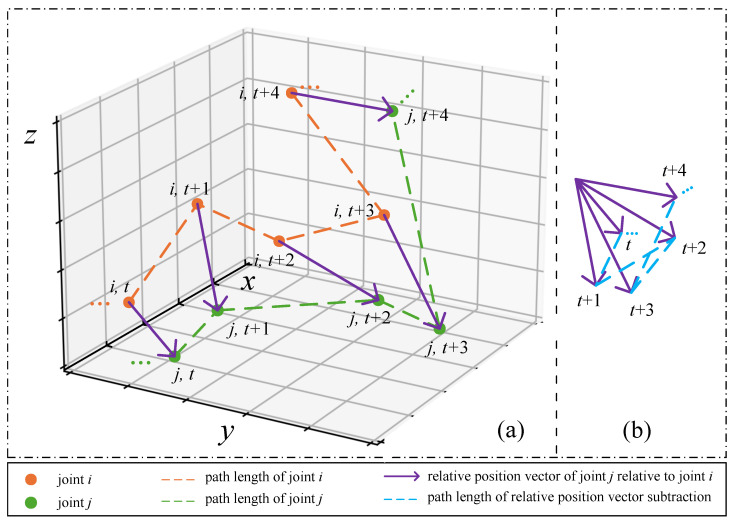
(**a**) The positions of joint *i* and joint *j* in different frames. (**b**) The relative position vectors of joint *j* relative to joint *i* in different frames.

Taking the *x* axis dimension as an example, the formula for calculating the inter-joint interaction intensity $E_{x}$ is as follows:

(12) $$\left\{\begin{array}{c} m_{ij}=X\left[1,:,j\right]-X\left[1,:,i\right]\\ M_{x}\left[:,i,j\right]=\left\{m_{ij}|i,j=1,2,\cdots ,V\right\}\\ E_{x}=N_{2}\left(\sum_{t=1}^{T-1}\left|M_{x}\left[t+1,:,:\right]-M_{x}\left[t,:,:\right]\right|\right) \end{array}\right.$$

where *X* refers to the original skeleton sequence, $N_{2}$ denotes the random walk normalization operation, |·| represents taking the absolute value, and $M_{x}\left[:,i,j\right]$ is the vector of joint *j* relative to joint *i* and it refers to the purple arrow in Figure 4, $M_{x}\in R^{T\times V\times V}$, $E_{x}\in R^{V\times V}$.

As shown in Figure 4a, the red dashed line and the green dashed line represent the trajectories of joint *i* and joint *j*, respectively. By adding their respective components on the *x*-axis, we obtain the path length of *x*-axis for joints *i* and *j*, which corresponds to $D_{x}$ in Equation (11). The purple arrow indicates the position vector of joint *j* relative to joint *i* in different frames. As shown in Figure 4b, the blue dashed line represents the trajectory obtained by subtracting the relative position vector of joint *j* relative to joint *i* in adjacent frames. We take the difference in the *x*-axis components and then sum these magnitudes to obtain the difference of joint *j* relative to joint *i*, which corresponds to $E_{x}$ in Equation (12).

The resulting matrix $E_{x}$ from this computation is a symmetric matrix with its main diagonal elements equal to zero. This is because $m_{ij}=m_{ji}=0$ and $|m_{ij}\left|=\right|m_{ji}|$ in Equation (12). In the node aggregation operation of GCN, more active nodes with more effective features should be assigned higher weights. We consider that the simple use of the symmetric matrix $E_{x}$ as a replacement for the original adjacency matrix might not be appropriate. When the interaction intensity between nodes *i* and *j* is high, that does not mean that both nodes *i* and *j* are equally active. To distinguish this, a more accurate relative weight between each pair of nodes can be obtained by multiplying the relative interaction matrix $E_{x}$ by the activity values of each node. This process better reflects the weight relationship between each pair of nodes. The computation is defined in Equation (13):

(13) $$R_{x}=N_{3}\left\{E_{x}\left[i,:\right]\times D_{x}\left[i\right]|i=1,2,\cdots ,V\right\}$$

where $N_{3}$ denotes the random walk normalization operation.

#### 3.3.2. Part 2 (*A*)

The computation of matrix *A* is inspired by the self-attention mechanism [32]. The input has a shape of $C_{in}\times T\times V$. As shown in Figure 3, the input is first passed through two separate 1×1 convolutional layers serving as embedding functions, resulting in two feature branches of shape $C_{e}\times T\times V$. These two feature branches then undergo average pooling along the temporal dimension. After pooling, the first feature branch is reshaped to $C_{e}\times V\times 1$, and the second is reshaped to $C_{e}\times 1\times V$. Using broadcasting, element-wise subtraction is performed between the two branches, followed by a tanh activation. This subtraction operation enables the module to capture localized joint-wise differences, while the tanh activation further preserves the directional characteristics of these deviations, allowing the model to encode both the magnitude and direction of local relational changes. Finally, average pooling is applied along the channel dimension to obtain matrix *A*.

The coefficient α in Equation (10) is initialized to 0. This design allows the model to rely primarily on the physically interpretable components (*R* and *C*) at the early training stage, while α is treated as a learnable parameter and is gradually adjusted through backpropagation to determine the effective contribution of matrix *A*. The computation of *A* is defined by Equation (14) as follows:

(14) $$A=pool_{c}\left(\tanh\left(\left(pool_{t}\left(f_{in}\cdot W_{1}\right)\right)-\left(pool_{t}\left(f_{in}\cdot W_{2}\right)\right)\right)\right)$$

where $W_{1}$ and $W_{2}$ are the parameters of the embedding functions, $pool_{t}$ represents temporal average pooling, and $pool_{c}$ represents channel average pooling.

#### 3.3.3. Part 3 (*B*)

As shown in Figure 3, the computation of matrix *B* follows a similar procedure to that of matrix *A*, except that the subtraction operation is replaced by a matrix multiplication operation, and the tanh activation is replaced by a Softmax function. Through the Softmax-normalized dot-product interaction, matrix *B* captures global relational dependencies by measuring the overall affinity between joints across the entire spatial dimension. Similarly, the coefficient β in the computation of *B* is initialized to 0 and is updated during training. This enables the network to adaptively learn the appropriate influence of the global correlation pathway represented by matrix *B*, without introducing unstable interactions at initialization. The calculation of *B* is given by Equation (15):

(15) $$\begin{array}{cc} B & ={\mathrm{pool}}_{c}\left(\mathrm{Softmax}\left({\mathrm{pool}}_{t}\left(f_{\mathrm{in}}\cdot W_{1}\right)\right)\right.\\ \; & \;\left.\times {\mathrm{pool}}_{t}\left(f_{\mathrm{in}}\cdot W_{2}\right)\right) \end{array}$$

where $W_{1}$ and $W_{2}$ are the parameters of the embedding functions, $pool_{t}$ represents temporal average pooling, and $pool_{c}$ represents channel average pooling.

#### 3.3.4. Part 4 (*C*)

Taking the *x* axis dimension as an example, as the matrix $E_{x}$ in Equation (12) is a symmetric matrix with zero elements along its main diagonal, the resulting matrix $R_{x}$ in Equation (13) also has zero diagonal elements. If $R_{x}$ is solely utilized as the weight matrix for node aggregation, it might result in inadequate attention being paid to the node itself. As a supplement, we introduce a matrix *C* and initialize it as an identity matrix to represent the self-connections of each node. To further enhance the model’s adaptability, the elements of matrix *C* are permitted to undergo continuous updates during the model training process. This allows the node relationship matrix to be adaptively adjusted, thereby more effectively capturing the dynamic changes in relationships between nodes across various actions.

### 3.4. MSTFF

**Figure 5:**
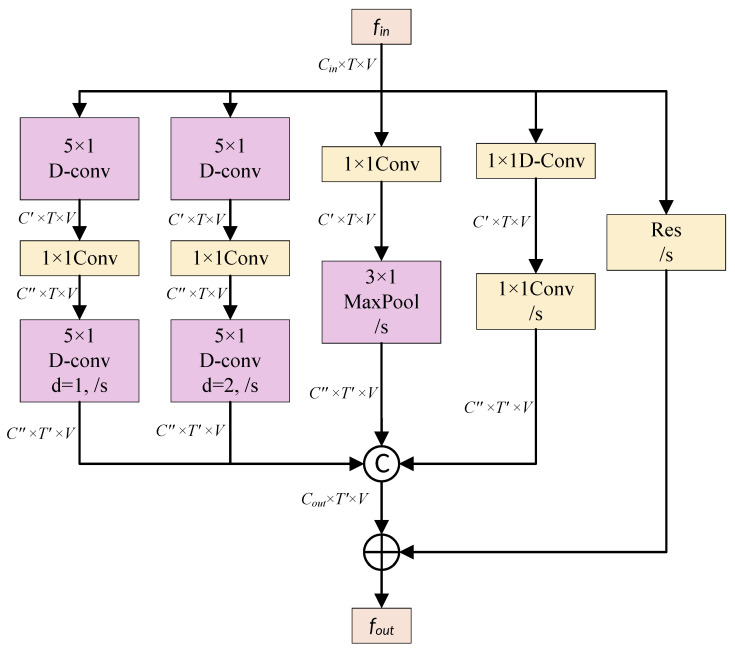
The structure of the MSTFF module, where D-conv represents depthwise convolution, d indicates the dilation rate in dilated convolutions, /s signifies a temporal stride of s, ⓒ denotes concatenation along the channel dimension, and ⊕ represents element-wise addition.

The MSTFF module is designed to extract and fuse temporal features of skeleton data across different time scales, capturing more comprehensive motion pattern information. The input and output channel dimensions of this module remain consistent. The overall structure consists of four parallel information extraction branches, each utilizing different convolution and pooling operations to extract features, thereby achieving multi-scale feature fusion. The structure of the MSTFF module is shown in Figure 5.

The four parallel information extraction branches are designed as follows. Branch 1 initiates with grouped convolution to extract temporal features, reducing the input channels by half, and subsequently applies pointwise convolution to further decrease the channels to one-fourth of the original count. It concludes with depthwise separable convolution to capture finer-grained temporal features. Branch 2 follows a similar pattern but distinguishes itself by setting the dilation rate of its final depthwise separable convolution to 2, enabling the capture of longer temporal dependencies and enhancing the extraction of long-term features. Branch 3 directly reduces the channel count to one-fourth using pointwise convolution and employs max pooling to extract global temporal information, highlighting salient features in the temporal dimension and enriching the diversity of feature representations. Lastly, Branch 4 begins with grouped convolution reducing the input channels by half, followed by pointwise convolution halving them again, effectively minimizing channel dimensions while preserving spatial information, thus improving computational efficiency.

The outputs of these four branches are then concatenated in the channel dimension and combined with the original input using a skip connection to produce the final output. The MSTFF module leverages parallel convolution and pooling operations to extract and fuse key features of skeleton data at different temporal scales. The model’s multi-branch architecture equips it with diverse feature representation capabilities, subsequently improving the recognition of intricate action patterns.

### 3.5. CEST-JAtt

Many existing attention modules for skeleton-based action recognition typically focus on a single dimension. For example, Part-Att [12] emphasized the information in the spatial dimension, STGR [37] targeted that in the temporal dimension, and ST-TR [33] employed self-attention mechanisms separately in the spatial and temporal dimensions to extract global information. Although STC-Att [36] introduced attention in three dimensions, including space, time, and channel, it did so by concatenating three single-dimensional attention mechanisms. It was not until the introduction of ST-JointAtt [13] that simultaneous attention to both temporal and spatial dimensions was achieved. To comprehensively consider the information in the three dimensions more efficiently, a novel attention module, the CEST-JAtt module, is designed in this paper. This module is designed to jointly identify the most informative joints and frames within a skeleton sequence while assigning different weights to different channels to enhance the representation capacity in the channel dimension.

**Figure 6:**
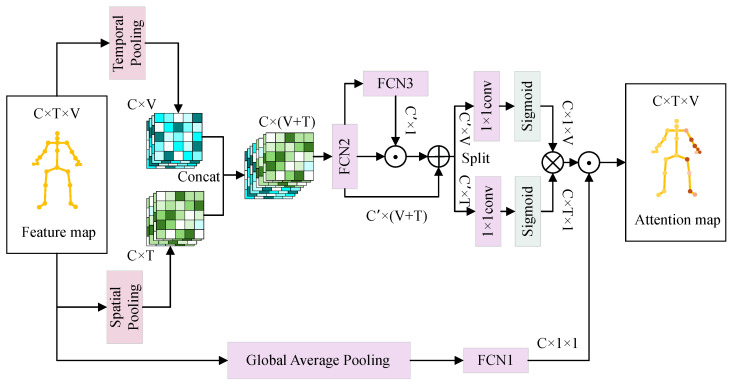
The structure of the CEST-JAtt module, where *C*, *T*, and *V* denote the number of input channels, frames, and joints, respectively; ⊕ represents concatenation operation; ⊙ indicates element-wise multiplication; and ⊗ denotes channel-wise matrix multiplication.

The architecture of the CEST-JAtt module is illustrated in Figure 6, and its computational process is divided into two branches. In the first branch, the input features undergo average pooling along the frame and joint dimensions. The resulting pooled feature vectors are concatenated and then passed through a fully connected layer (FCN2), for information compression and integration. Subsequently, dimensionality reduction and expansion operations are performed to compute channel-wise weights, as represented by FCN3. These computed weights are multiplied with the compressed features, and a residual mechanism is employed to reintroduce the original compressed features. Separate convolutional operations are used to compute attention scores for the temporal and joint dimensions, with the resulting weight matrices being transposed and multiplied to produce the output of the first branch. In the second branch, global pooling is applied to the input features, followed by two linear transformations corresponding to FCN1, to compute channel-specific attention scores. This results in the output of the second branch. Finally, the outputs of the two branches are multiplied to generate a unified attention score for the entire sequence.

The proposed CEST-JAtt module can be formulated as follows:

(16) $$\left\{\begin{array}{c} FCN1\left(X\right)=\varphi (\phi \left(X\cdot W_{1}\right)\cdot W_{2})\\ FCN2\left(X\right)=\theta (BN\left(X\cdot W_{3}\right))\\ FCN3\left(X\right)=\varphi (\phi \left(X\cdot W_{4}\right)\cdot W_{5})\\ f_{inner1}=FCN2\left(pool_{t}\left(f_{in}\right)⊕pool_{s}\left(f_{in}\right)\right)\\ f_{inner2}=f_{inner1}⊙FCN3\left(f_{inner1}\right)+f_{inner1}\\ f_{inner3}=\varphi \left(f_{inner2}\cdot W_{6}\right)⊗\varphi \left(f_{inner2}\cdot W_{7}\right)\\ f_{inner}^{\prime }=FCN1\left(pool_{g}\left(f_{in}\right)\right)\\ f_{out}=f_{inner3}⊙f_{inner}^{\prime } \end{array}\right.$$

where ϕ(·), φ(·), and θ(·) denote the ReLU, Sigmoid, and HardSwish activation function, respectively; *X* refers to the input for FCN1, FCN2, and FCN3; $f_{in}$ and $f_{out}$ represent the input and output feature maps of the attention module, respectively; $pool_{t}$ indicates temporal pooling; $pool_{s}$ indicates spatial pooling; $pool_{g}$ indicates global spatiotemporal pooling; ⊕ represents concatenation operation; ⊙ denotes element-wise multiplication; and ⊗ denotes channel-wise matrix multiplication.

## 4. Experiments

In this section, a great number of experiments are conducted by using the proposed LI-AGCN on three large public datasets: NTU RGB+D [41], NTU RGB+D 120 [42], and UAV-Human [43]. The effectiveness of our method is validated through ablation experiments and extensive comparisons with state-of-the-art (SOTA) methods.

### 4.1. Dataset

#### 4.1.1. NTU RGB+D

This dataset contains 60 action classes with 56,680 samples. Two benchmarks are provided: (1) Cross-Subject (X-Sub); (2) Cross-View (X-View).

#### 4.1.2. NTU RGB+D 120

This is an extension of the NTU RGB+D dataset, containing 120 action classes with 114,480 samples. Two benchmarks are provided: (1) Cross-Subject (X-Sub120); (2) Cross-Set (X-Set120).

#### 4.1.3. UAV-Human

This dataset covers 155 action classes with 67,428 samples. Two benchmarks are provided: (1) Cross-Subject-v1 (CSv1); (2) Cross-Subject-v2 (CSv2).

### 4.2. Implementation Details

In our experimental setup, the model undergoes training for a maximum of 70 epochs. The optimization process employs stochastic gradient descent (SGD) with a Nesterov momentum of 0.9 and a weight decay coefficient of 0.0001. The learning rate is initialized at 0.1. A linear warm-up [18] strategy is applied during the first 10 epochs, where the learning rate is gradually increased from near zero to the initial value of 0.1; this approach helps stabilize the initial phase of training by preventing large gradient updates from disrupting the randomly initialized model parameters. After the warm-up phase, the learning rate is adjusted using a cosine annealing schedule, which reduces the learning rate gradually, following a cosine curve for the remaining 60 epochs. This combination allows for stable convergence and refined tuning in the later stages of training. The current hyperparameter settings are designed to ensure training stability, and the model has demonstrated favorable convergence under this configuration. All experiments are implemented using the PyTorch 1.12.1 deep learning framework and are executed on an NVIDIA RTX 4090 GPU to accelerate computational throughput.

### 4.3. Comparison with SOTA Methods

To demonstrate the comprehensive performance advantages of LI-AGCN, we compare it with several SOTA methods on three datasets: NTU RGB+D, NTU RGB+D 120, and UAV-Human. The comparison results on the NTU datasets are presented in Table 1 and Table 2, respectively. As shown in Table 1, LI-AGCN achieves higher classification accuracy than typical models despite having a significantly lower parameter count, e.g., with only 1/17 of the parameter count of ST-GCN [11] and 1/53 of that of AS-GCN [44]. When compared to high-accuracy models, LI-AGCN demonstrates that there is a slight gap in accuracy. For instance, both MS-G3D [22] and CTR-GCN [27] achieve an accuracy of more than 91% on the X-sub benchmark of the NTU RGB+D dataset and an accuracy of more than 96% on the X-view benchmark of the NTU RGB+D dataset. Furthermore, it outperforms most SOTA methods in terms of parameter efficiency. For instance, compared to the lightweight model ResGCN-N51 [12], LI-AGCN uses only a quarter of its parameter count while achieving higher accuracy across all benchmarks on the NTU datasets. Furthermore, when compared to EfficientGCN-B0 [13], LI-AGCN achieves comparable classification accuracy with approximately half the number of parameters. Notably, LI-AGCN also performs excellently on the UAV-Human dataset. As shown in Table 2, LI-AGCN achieves comparable or even higher accuracy than other SOTA methods with only 0.18 million parameters and 1.60 giga FLOPs, demonstrating its effectiveness in complex environments. Overall, LI-AGCN not only achieves reliable classification accuracy but also excels with a remarkably low number of parameters, highlighting its potential for efficient and scalable applications.

### 4.4. Ablation Studies

In this section, we analyze the contributions of several key components in the proposed LI-AGCN model. The evaluation will focus on several key components: the selection of TSFE modules, temporal feature extraction modules, and attention modules; feature selection in data preprocessing; and the selection of the channel reduction factor in MSFE module. All experiments are conducted on the NTU RGB+D dataset.

#### 4.4.1. Comparisons of Different TSFE Modules

This subsection evaluates the effectiveness of different TSFE modules. The ASR-AGC module is employed as the TSFE module in the Initial block, as shown in Figure 2. To further validate the contribution of each component within ASR-AGC module, ablation studies are conducted by sequentially removing the SGC module, matrix *R*, matrix *C*, matrix *A*, and matrix *B* separately to demonstrate their respective roles. For broader comparison, we also replace TSFE module with spatial modules from CTR-GCN [27], ShiftGCN++ [52], and 2s-AGCN [26]. Results on the NTU RGB+D X-Sub benchmarks are summarized in Table 3, demonstrating that the complete ASR-AGC module yields the best performance, thereby confirming its effectiveness.

#### 4.4.2. Necessity of Data Preprocessing

The CIB architecture (Figure 2) is introduced in Section 3.2 and includes data preprocessing steps that integrate joint positions (P), velocities (V), and bone features (B) from raw skeleton sequences. To verify the importance of considering all three features, we conduct experiments with different feature combinations. The results, as shown in Table 4, reveal that the model incorporating all three features (PVB) achieves the highest accuracy while maintaining nearly the same parameter count as models using a single feature. These results validate the effectiveness of the CIB architecture.

#### 4.4.3. Comparisons of Different MSFE Modules

In the LW block (Figure 2), we employ the SGC module as the MSFE module. To achieve a lightweight design, a depthwise separable convolution is added in front of the SGC module to reduce the number of channels by a factor *r*, effectively lowering the number of channels by *r* times. In LI-AGCN, we set r=2. To further validate this design, we experiment with r=4, r=8, and r=1, respectively. As shown in Table 5, the results demonstrate that r=2 achieves the highest accuracy, confirming it as the optimum setting.

#### 4.4.4. Comparisons of Different Temporal Feature Extraction Modules

As shown in Figure 2, both the primary and auxiliary temporal feature extraction modules in LI-AGCN employ the proposed MSTFF module. To validate its effectiveness, we replace MSTFF with other temporal feature extraction modules, including BasicLayer [11], BottleLayer [12], EpSepLayer, and SGLayer (EfficientGCN [13]). The results, presented in Table 6, show that MSTFF module achieves the highest accuracy with the lowest parameter count and the least FLOPs, demonstrating its superiority.

#### 4.4.5. Comparisons of Different Attention Modules

To enhance feature extraction and modeling capabilities, the proposed CEST-JointAtt module is embedded into convolutional blocks. We compare CEST-JointAtt with ST-JointAtt [13], ChannelAtt [12], FrameAtt [12], JointAtt [12], STCAtt [36], and PartAtt [12]. Results in Table 7 indicate that CEST-JointAtt achieves significantly higher accuracy with comparable parameter count and computational complexity (FLOPs), validating its effectiveness.

#### 4.4.6. Incremental Analysis of Module Contributions to the Baseline Model

To comprehensively evaluate the individual and joint contributions of the ASR-AGC, MSTFF, and CEST-JAtt modules, we developed a structured ablation study. We first established a baseline model that adopts the CIB-based architecture, where the number of layers and the channel configurations follow the design illustrated in Figure 2. In this baseline, the TSFE module employs a simple SGC module, temporal information is captured using a standard 1D convolution, and no attention mechanism is applied.

Building upon this baseline, we gradually introduced the proposed modules in a controlled manner: (1) replacing only the TSFE module with ASR-AGC module; (2) replacing only the temporal modeling component with MSTFF module; and (3) adding only the CEST-JAtt module. We further evaluated all pairwise combinations of these modules to examine their interactions. As shown in Table 8, each individual module provides a noticeable performance gain over the baseline, while pairwise combinations lead to further improvements. Ultimately, integrating all three modules simultaneously yields the best overall performance, substantially surpassing both the baseline and all partially enhanced variants. These results confirm not only the effectiveness of each proposed module but also the synergistic benefits arising from their joint integration.

### 4.5. Visualization of Results

**Figure 7:**
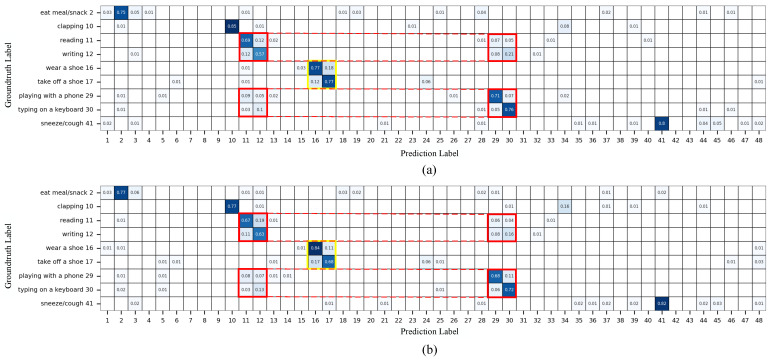
(**a**,**b**) Confusion matrices showing failure actions (with accuracy less than 79% on the X-sub benchmark) for LI-AGCN and EfficientGCN-B0, respectively. In these matrices, the numbers on the coordinate axes represent the indexes of each action category. The red and yellow rectangles highlight two groups of similar actions. Grid squares without numbers indicate a probability of 0.

We conduct visualization experiments to analyze the performance of LI-AGCN on the X-Sub benchmark and generate a confusion matrix to evaluate the model’s action recognition accuracy. Although LI-AGCN receives promising results on the large-scale datasets, certain actions, such as reading, writing, typing on a keyboard, and using a mobile phone, have relatively lower recognition rates. These actions are frequently misclassified among each other, primarily because their differences lie in subtle hand movements. The NTU skeleton dataset includes only three joints on each hand, and only the subtle movements of hand joints occur for these actions, making it a challenge for the model to accurately learn and distinguish these behaviors. Similarly, taking off shoes and putting on shoes are also frequently misclassified due to the limited number of joints in the foot region and the highly similar joint movement patterns between these two actions. We compare the accuracy of these easily misclassified action categories with EfficientGCN-B0 [13], and the comparison results are shown in Figure 7. The results indicate that EfficientGCN-B0 has a significant accuracy advantage in the actions of writing and putting on shoes, while LI-AGCN performs better in the other several actions.

**Figure 8:**
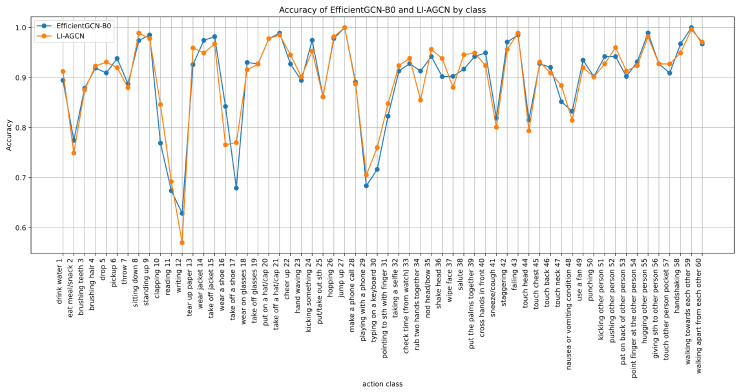
The line chart of action accuracy of LI-AGCN for each action on X-Sub benchmark.

To provide a more intuitive representation of the recognition accuracy for each action category, we plot a curve of accuracy and compare it with EfficientGC-B0, as shown in Figure 8. The two curves visually exhibit the recognition rate of each action for both LI-AGCN and EfficientGC-B0, and highlight the comparative performance among different categories. It can be observed that the accuracy rates of the two models for various action categories are quite similar.

**Figure 9:**
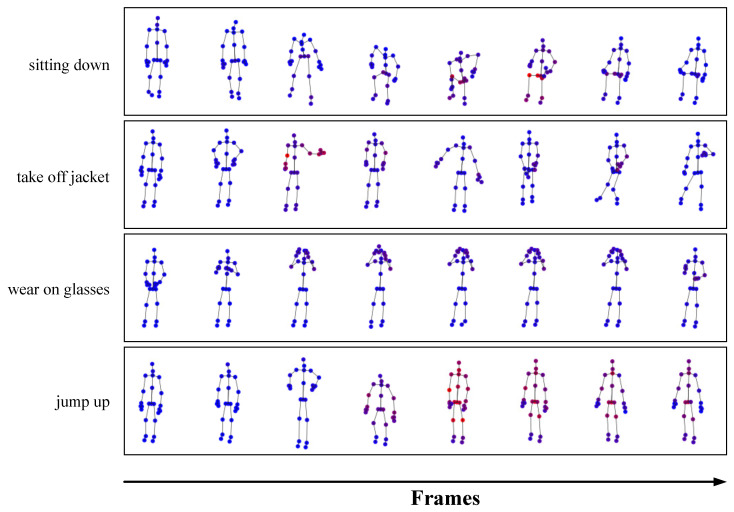
Activated joints of four action classes on the X-Sub benchmark. The solid red circles indicate activated joints and the blue ones indicate inactivated joints.

In addition, we employ the class activation mapping (CAM) technique [53] to compute and visualize joint activation maps for skeleton sequences. As shown in Figure 9, we provide class activation maps for four actions in the NTU dataset: sitting down, taking off a jacket, putting on glasses, and jumping up. These images are composed of the frames extracted randomly in temporal order from the original sequence. These maps not only visualize the predicted action classes but also display the activated joints across different frames in the action samples, with varying degrees of red indicating the activation intensity of each joint. The joint activation maps effectively identify the specific joints that play a critical role in performing the actions, offering valuable insights for further model optimization and interpretability.

## 5. Conclusions

This paper proposes a model named LI-AGCN to address key challenges in skeleton-based action recognition. This model enhances the extraction of spatial initial information, effectively capturing the relationships between any two joints. Simultaneously, during the extraction of temporal information, it achieves efficient results by considering lightweight design and multi-scale strategies. Furthermore, we effectively combine the coordinate-based early fusion strategy with the attention mechanism, significantly improving the model’s accuracy. Extensive experiments on the NTU RGB+D, NTU RGB+D 120, and UAV-Human datasets demonstrate that LI-AGCN achieves comparable or even higher recognition accuracy than SOTA methods while significantly reducing parameter count and FLOPs. Notably, LI-AGCN’s performance on the UAV-Human dataset further validates its effectiveness in complex and dynamic environments. Ablation studies confirm the effectiveness of each module and their contributions to the overall model performance, providing strong theoretical support.

In future work, we will continue improving the LI-AGCN architecture to enhance its ability to recognize subtle actions and explore the integration of multi-modal data (e.g., RGB and depth) to further strengthen generalization. Additionally, given the strict computational and power constraints of practical edge devices, we will focus on real-device deployment and engineering optimization of LI-AGCN, including evaluations of latency, memory usage, and power consumption on mobile and embedded platforms.

## Figures and Tables

**Table 1:** Comparisons with SOTA methods on NTU RGB+D and NTU RGB+D 120 in accuracy, FLOPs, and number of parameters.

Model	Param. (M)	FLOPs (G)	NTU RGB+D (%)		NTU RGB+D 120 (%)	
			X-Sub	X-View	X-Sub120	X-Set120
ST-GCN [11]	3.10 *	16.32 *	81.5	88.3	70.7 *	73.2 *
AS-GCN [44]	9.50 *	26.76 *	86.8	94.2	77.9 *	78.5 *
2s-AGCN [26]	6.94 *	37.32 *	88.5	95.1	82.5 *	84.2 *
DGNN [45]	26.24	-	89.9	96.1	-	-
ResGCN-N51 [12]	0.77	5.41 *	89.1	93.5	84.0	84.2
1s-ShiftGCN [46]	0.69	2.50	87.8	95.1	80.9	83.2
MS-G3D [22]	6.40 *	48.88 *	91.5	96.2	86.9	88.4
MS-AAGCN [36]	15.65	-	90.0	96.2	-	-
MST-GCN [47]	12.00 *	64.14 *	91.5	96.6	87.5	88.8
CTR-GCN [27]	1.46	1.97	92.4	96.8	88.9	90.6
EfficientGCN-B0 [13]	0.29	2.73	90.2	94.9	86.6	85.0
IA-ASGCN [16]	3.84	-	90.5	95.8	85.4	87.4
Js STF-Net [48]	1.70	7.50	88.8	95.0	-	-
VA-DGCN [49]	14.46	31.56	92.6	96.9	89.1	90.9
LC-AGCN [14]	0.56	-	89.3	94.0	84.2	84.9
Mss-AGCN [25]	0.87	**1.11**	90.4	95.5	85.9	87.3
SAN-GCN [24]	2.41	-	90.0	94.7	85.0	86.6
MDR-GCN [50]	1.30	15.30	**92.8**	**97.2**	**89.8**	**91.3**
LI-AGCN (ours)	**0.18**	1.60	90.0	94.5	85.5	85.0

*: These results are implemented based on the released codes.

**Table 2:** Comparisons with SOTA methods on CSv1 benchmark and CSv2 benchmark of UAV-Human in accuracy and number of parameters.

Model	Param. (M)	CSv1 (%)	CSv2 (%)
ST-GCN [11]	3.10	30.3 *	56.1 *
DGNN [45]	26.24	29.9	-
1s-ShiftGCN [46]	0.69	38.0 *	67.0 *
2s-AGCN [26]	6.94	34.5 *	66.7 *
CTR-GCN [27]	1.46	**43.4** *	-
EfficientGCN-B0 [13]	0.29	39.2 *	63.2 *
STGPCN [51]	1.70	41.5	67.8
LI-AGCN (ours)	0.18	**43.4**	**69.3**

*: These results are implemented based on the released codes.

**Table 3:** Comparisons of different TSFE modules on NTU RGB+D X-Sub benchmark in accuracy, FLOPs, and number of parameters.

Model	Param. (M)	FLOPs (G)	X-Sub (%)
CTR-GCN [27]	0.21	1.71	89.06
ShiftGCN++ [52]	0.18	1.47	88.68
2s-AGCN [26]	0.18	1.54	89.20
ASR-AGC wo/SGC	0.17	1.51	89.29
ASR-AGC wo/R	0.18	1.60	89.51
ASR-AGC wo/C	0.18	1.60	89.86
ASR-AGC wo/A	0.18	1.60	89.72
ASR-AGC wo/B	0.18	1.60	89.82
ASR-AGC	0.18	1.60	**90.03**

**Table 4:** Comparisons of different input features on X-Sub benchmark of NTU RGB+D in accuracy, FLOPs, and number of parameters.

Input	Param. (M)	FLOPs (G)	X-Sub (%)
P	0.18	1.52	88.26
V	0.18	1.52	87.46
B	0.18	1.52	88.55
PV	0.18	1.56	88.80
VB	0.18	1.56	89.52
PB	0.18	1.56	89.41
PVB	0.18	1.60	**90.03**

**Table 5:** Comparisons of different channel reduction factor in MSFE module on X-Sub benchmark of NTU RGB+D in accuracy, FLOPs, and number of parameters.

r	Param. (M)	FLOPs (G)	X-Sub (%)
1	0.19	1.63	88.73
2	0.18	1.60	**90.03**
4	0.16	1.28	89.29
8	0.15	1.16	89.28

**Table 6:** Comparisons of different temporal extraction modules on X-Sub benchmark of NTU RGB+D in accuracy, FLOPs, and number of parameters.

Module	Param. (M)	FLOPs (G)	X-Sub (%)
BasicLayer [11]	0.32	2.97	89.11
BottleLayer [12]	0.23	2.07	89.32
SGLayer [13]	0.19	1.77	89.46
EpSepLayer [13]	0.29	2.97	89.48
MSTFF	0.18	1.60	**90.03**

**Table 7:** Comparisons of different attention modules on X-Sub benchmark of NTU RGB+D in accuracy, FLOPs, and number of parameters.

Attention	Param. (M)	FLOPs (G)	X-Sub (%)
None	0.12	1.59	88.15
ChannelAtt [12]	0.14	1.59	88.53
FrameAtt [12]	0.12	1.59	88.71
JointAtt [12]	0.13	1.59	88.84
PartAtt [12]	0.19	1.59	88.54
ST-JointAtt [13]	0.16	1.59	89.52
STCAtt [36]	0.15	1.59	89.10
CEST-JAtt	0.18	1.60	**90.03**

**Table 8:** Comparisons of different module combinations on X-Sub benchmark of NTU RGB+D in accuracy, FLOPs, and number of parameters. (✓ indicates that the corresponding module is used, while × indicates it is not used.)

ASR-AGC	MSTFF	CEST-JAtt	Param. (M)	FLOPs (G)	X-Sub (%)
×	×	×	0.25	2.84	87.15
✓	×	×	0.26	2.95	87.98
×	✓	×	0.12	1.48	88.03
×	×	✓	0.31	2.85	88.89
✓	✓	×	0.12	1.59	88.15
×	✓	✓	0.17	1.49	89.42
✓	×	✓	0.32	2.97	89.11
✓	✓	✓	0.18	1.60	**90.03**

## Data Availability

We will provide links to the three datasets used in our experiments. NTU RGB+D 60 and NTU RGB+D 120: https://rose1.ntu.edu.sg/dataset/actionRecognition/, (accessed on 2 November 2024). UAV Human: https://sutdcv.github.io/uav-human-web/, (accessed on 10 November 2024).
